# Citrus flavanones (naringenin, hesperidin, and hesperetin) in oral and gastric carcinogenesis: mechanistic insights into oxidative stress, cell death, epigenetic regulation, non-coding RNAs, and tumor microenvironment remodeling

**DOI:** 10.3389/fcell.2026.1890060

**Published:** 2026-08-12

**Authors:** Ruiting Liang, Biying Wang, Yan Liu

**Affiliations:** 1 Department of Oral and Maxillofacial Surgery, The Ninth Medical Center of the Chinese People’s Liberation Army General Hospital, Beijing, China; 2 Department of Oral and Maxillofacial Surgery, Chinese People’s Liberation Army General Hospital, Beijing, China

**Keywords:** chemoprevention, citrus flavonoids, epigenetic regulation, gastric cancer, hesperetin, naringenin, oral cancer

## Abstract

Flavonoids derived from citrus fruits, including naringenin, naringin, hesperetin, and hesperidin, represent a class of dietary bioactives with potential chemopreventive properties against oral cancer and gastric cancer (GC). However, previous reviews have largely focused on isolated molecular pathways or single cancer types, lacking an integrated comparison of oral and gastric carcinogenesis within a unified mechanistic framework. Preclinical investigations using chemical carcinogenesis models consistently show that these compounds attenuate oxidative stress, restore cellular redox balance, and modulate critical regulators of tumor progression through interconnected redox-sensitive signaling pathways. Nanoparticle-based delivery enhances bioavailability, tissue targeting, and pharmacokinetics, amplifying their therapeutic efficacy. In oral carcinoma models, flavonoids suppress tumor growth and enhance chemosensitivity, whereas in GC they inhibit malignant progression and metastatic behavior. Additionally, these compounds influence the tumor microenvironment (TME) by modulating immune surveillance, suppressing PD-L1 expression, restraining angiogenesis, and interacting with microbial and inflammatory pathways. Epigenetic and non-coding RNA mechanisms, including DNA methylation, microRNA, and long non-coding RNA regulation, further contribute to their anticancer activity. Unlike previous reports, this review integrates intracellular signaling, TME modulation, epigenetic regulation, and nanodelivery strategies across both oral and GC models, providing a unified multi-layered mechanistic framework. Collectively, these findings delineate a multi-layered mechanistic framework for citrus flavonoids, supporting their promise as adjunctive chemopreventive agents while emphasizing the necessity for translational and clinical validation.

## 1 Introduction

Citrus fruits are among the most extensively cultivated and consumed crops worldwide, prized not only for their economic and nutritional value but also for the richness of their bioactive constituents (Sharma et al., 2019). Within these fruits, flavonoids especially the subclass of flavanones such as naringenin (NAR), naringin, and hesperidin (Hsp), have emerged as compounds of considerable interest due to their diverse biological activities. Traditionally recognized for their antioxidant and anti-inflammatory properties, recent investigations have revealed their broader capacity to modulate systemic metabolic processes, immune responses, and cellular functions (Manthey and Grohmann, 1996; Yang et al., 2022). Notably, byproducts of citrus processing, including peels, pith, and seeds, are particularly enriched in these compounds and can be valorized to produce concentrated flavonoid extracts with enhanced functional potential (Sharma et al., 2019; Manthey and Grohmann, 1996). The bioavailability and chemical form of flavanones critically determine their physiological effects. Glycosylated forms, such as Hsp and narirutin, require enzymatic hydrolysis to release absorbable aglycones, whereas compounds like NAR are more readily absorbed in the small intestine (Nielsen et al., 2006; Silveira et al., 2014; Vallejo et al., 2010). Human pharmacokinetic studies demonstrate that both the concentration and solubility of flavanones, alongside enzymatic modifications, strongly influence plasma exposure and tissue distribution, ultimately modulating systemic biological activity (Nielsen et al., 2006; Park et al., 2011). Furthermore, co-consumption with dietary matrices or combinations of flavonoids can interact with intestinal microbiota and metabolic pathways, highlighting the complexity of translating *in vitro* efficacy into *in vivo* bioactivity (Wang M. et al., 2021; Cichon et al., 2025).

Beyond their established systemic bioactivities, increasing evidence indicates that citrus flavanones directly modulate molecular pathways relevant to carcinogenesis, including redox homeostasis, inflammatory signaling, cell survival, and tumor–host interactions (Yang et al., 2022; Parhiz et al., 2015; Hao et al., 2025; Liang HQ. et al., 2025; Pinho-Ribeiro et al., 2016; Zhang et al., 2021; Smeriglio et al., 2023). Crucially, the anticancer potential of citrus flavanones has been substantiated across multiple preclinical models. Both NAR and Hsp can suppress cancer cell proliferation, induce cell cycle arrest, and trigger apoptosis in various malignancies, including gastric, and other epithelial cancers. Citrus peel, often discarded as industrial waste, is particularly rich in flavanones and polymethoxylated flavones, offering a sustainable and potent source of chemopreventive compounds. These findings support the notion that dietary citrus flavanones may function not only as preventive nutrients but also as adjunctive agents in cancer chemoprevention strategies (Manthey and Guthrie, 2002; Koolaji et al., 2020; Nair et al., 2018; Frydoonfar et al., 2003). However, existing literature often lacks an integrated framework that simultaneously connects intracellular signaling, tumor microenvironment (TME) modulation, epigenetic regulation, and nanotechnology-based delivery across oral squamous cell carcinoma (OSCC) and gastric cancer (GC) models.

Accordingly, this review focuses on the comparative molecular mechanisms by which citrus flavanones influence OSCC and GC, integrating intracellular signaling, TME remodeling, epigenetic regulation, non-coding RNA networks, and nanodelivery strategies within a unified mechanistic framework.

## 2 Systemic immunomodulatory and antioxidant effects of citrus flavanones

Citrus-derived flavanones, including Hsp, naringin, and NAR, are bioactive polyphenols with well-documented roles in modulating oxidative stress and inflammatory responses at both systemic and tissue levels. Preclinical studies suggest that these compounds may contribute to immune regulation, redox balance, and maintenance of tissue homeostasis across multiple organ systems, supporting their potential relevance in dietary and therapeutic contexts (Miles and Calder, 2021; Gandhi et al., 2020). Across diverse preclinical disease models, Hsp, naringin, and NAR consistently attenuate inflammatory signaling and improve redox homeostasis, supporting their broad immunomodulatory potential (Jin et al., 2017). Mechanistically, these flavanones suppress pro-inflammatory cytokine production, regulate immune-cell activity, and modulate redox-sensitive pathways, including nuclear factor κB (NF-κB) and AMP-activated protein kinase (AMPK) signaling, although most supporting evidence derives from non-cancer experimental models (Jin et al., 2017; Zhu et al., 2026; Ge et al., 2022). Beyond immune modulation, flavanone distribution and bioavailability contribute significantly to their biological effects. After oral administration, NAR and its metabolites are detected in multiple organs, including the brain, heart, and lungs, indicating systemic exposure and tissue-specific activity of both parent compounds and conjugated forms (El Mohsen et al., 2004). Experimental studies further indicate that flavanones improve systemic inflammatory and antioxidant status following oral administration (Ferreira et al., 2016). These systemic observations suggest that citrus flavanones exert pleiotropic biological activities beyond cancer, although their relevance to oral and gastric carcinogenesis requires disease-specific investigation (Khan et al., 2020; Fast et al., 2019; Abas et al., 2025). Collectively, citrus flavanones exhibit pleiotropic immunomodulatory and antioxidant properties. However, most evidence remains preclinical, and further studies are required to clarify pharmacokinetics, bioavailability, tissue targeting, and long-term safety in humans. These findings provide a rationale for the subsequent discussion of flavanone pharmacokinetics, microbiota interactions, and formulation strategies in the following sections (Habauzit et al., 2011; Dadwal and Gupta, 2023).

## 3 Pharmacokinetics, microbiota modulation, and formulation strategies of citrus flavanones

Flavanones, including NAR, Hesperetin (HET), naringin, and Hsp, occur as both aglycone and glycoside forms, with their chemical structure being a major determinant of oral bioavailability. Aglycones such as NAR and HET are absorbed more readily in the small intestine, whereas glycosides including naringin and Hsp require hydrolysis by intestinal enzymes and the gut microbiota before efficient absorption can occur (Felgines et al., 2000; Bredsdorff et al., 2010). Following absorption, these compounds undergo extensive phase II metabolism, primarily through glucuronidation and sulfation, resulting in circulating glucuronide and sulfate metabolites that predominate over the parent aglycones (Wang et al., 2006; Kanaze et al., 2007; Orrego-Lagarón et al., 2015). Efflux transporters further limit systemic exposure by exporting flavanone conjugates back into the intestinal lumen, contributing to their relatively low oral bioavailability. Consequently, the pharmacological activity observed *in vivo* is likely mediated predominantly by circulating conjugated metabolites rather than by the parent compounds (Wang et al., 2006; Kanaze et al., 2007; Guo et al., 2022; Bai et al., 2020). The intestinal microbiota plays a central role in flavanone metabolism by converting glycosides such as naringin and Hsp into absorbable aglycones. Consequently, inter-individual differences in microbial composition can substantially influence flavanone bioavailability and metabolic profiles (Wu et al., 2024). Conversely, flavanones may also modulate gut microbial composition and intestinal homeostasis, suggesting a bidirectional interaction between dietary flavanones and the gut microbiota (Wu et al., 2024).

### 3.1 Nanoparticle-based delivery

Because poor aqueous solubility and extensive first-pass metabolism limit the systemic bioavailability of citrus flavanones, several nanoformulation strategies have been developed to improve their pharmacokinetic performance. Nanoencapsulation of NAR has been shown to improve cellular uptake and intracellular retention, thereby enhancing its biological activity in preclinical models (Elwan et al., 2025). Similarly, HET nanoparticles (HETNPs) formulations also enhance cytotoxic and pro-apoptotic effects compared with free HET in oral carcinoma models (Elmoghayer et al., 2024). In addition, chitosan–folate-coated solid lipid nanoparticles and cyclodextrin/chitosan-based formulations have been shown to improve flavanone solubility, prolong drug release, and enhance tissue delivery compared with their non-encapsulated counterparts (Elmoghayer et al., 2024; Pohl et al., 2017; Al-Kariti et al., 2025; Gurushankar et al., 2015). Collectively, these findings suggest that nanoformulations may improve flavanone delivery and biological activity; however, the available evidence remains predominantly preclinical, and direct pharmacokinetic comparisons in humans are still lacking.

### 3.2 Human intake and translational considerations

Human pharmacokinetic studies indicate that Hsp and naringin are generally well tolerated at doses commonly achieved through citrus-rich diets or dietary supplementation. Plasma exposure varies considerably depending on the formulation, food matrix, and inter-individual differences in gut microbiota composition (Manach et al., 2005; Testai and Calderone, 2017). Clinical studies have administered Hsp at doses of approximately 500–1,000 mg/day and naringin or NAR at approximately 300–600 mg/day without major safety concerns, although standardized therapeutic dosing has not yet been established (Testai and Calderone, 2017). Preclinical studies further suggest that optimized formulations retain the favorable safety profile of flavanones while enhancing tissue delivery and therapeutic potential (Fida, 2023; Yoshino et al., 2016; Li WS. et al., 2021). Although these findings support the translational potential of flavanones, current evidence remains insufficient to establish their clinical efficacy or define evidence-based dosing recommendations.

## 4 Immunomodulatory and epigenetic regulation by citrus flavanones

Citrus flavanones, particularly NAR, naringin, and Hsp, act as pleiotropic modulators of immune responses and epigenetic regulation across multiple preclinical models. These compounds regulate both innate and adaptive immunity by modulating cytokine production, enhancing T cell and natural killer (NK) cell activity, and promoting macrophage polarization toward anti-inflammatory phenotypes. In parallel, accumulating evidence suggests that flavanones can influence gene expression through epigenetic and post-transcriptional mechanisms, indicating an integrated role in cellular signaling and transcriptional control that may underlie their protective effects (Martínez et al., 2019; Moriguchi et al., 2002). Across preclinical models, flavanones consistently suppress inflammatory signaling, regulate oxidative stress, and modulate immune-cell function, providing a mechanistic basis for their potential anticancer activity (Zhang et al., 2024). Preclinical evidence further indicates that flavanones can remodel the tumor immune microenvironment. Nanoparticle-based formulations of Hsp and related compounds reduce pro-inflammatory cytokine secretion, suppress fibroblast-mediated matrix remodeling, and enhance cytotoxic T lymphocyte infiltration, thereby attenuating immunosuppressive conditions (Huang et al., 2024). Similarly, Hsp-loaded nanoparticles promote macrophage reprogramming toward an M2-like phenotype, reduce IL-6 and TNF-α expression, and inhibit pathological angiogenesis (Wang M. et al., 2026). Oral administration studies further confirm systemic immune modulation, including increased NK cell activity, CD8^+^ cytotoxic T lymphocyte (CTL) responses and interferon-gamma (IFN-γ) production without major effects on humoral immunity (Camps-Bossacoma et al., 2017). Collectively, these findings support a broader immunoregulatory role for citrus flavanones that may contribute to antitumor immune responses (Guan et al., 2025; Zhang et al., 2026). Experimental studies demonstrate that citrus flavanones regulate multiple microRNA (miRNA) involved in inflammation, oxidative stress, metabolism, and cell survival, highlighting their broad epigenetic regulatory capacity across diverse biological contexts (Li et al., 2016; Helmy et al., 2020; Fan et al., 2015; Li SH. et al., 2021; Zhao et al., 2020). Furthermore, Hsp regulates miRNA expression in cancer models by restoring miR-149 via epigenetic mechanisms involving DNA methyltransferase 1, while nanoformulated Hsp suppresses oncogenic miR-21 and miR-155, inducing apoptosis and cell cycle arrest in cancer cells (Tian et al., 2021; Magura et al., 2021). Collectively, citrus flavanones act at the interface of immunomodulation and epigenetic regulation by coordinating cytokine signaling, immune cell function, and miRNA-mediated gene regulation. These mechanisms provide a conceptual framework for understanding how citrus flavanones may influence oral and gastric carcinogenesis through coordinated regulation of immune and epigenetic pathways. Although preclinical evidence is strong, further studies are required to clarify tissue-specific targets, optimize delivery strategies, and validate translational relevance in human systems.

## 5 Molecular mechanisms and chemoprevention of citrus flavonoids in oral and GC

### 5.1 NAR and HET in 7,12-dimethylbenz[a]anthracene (DMBA)-induced oral carcinogenesis: chemopreventive insights

**FIGURE 1:**
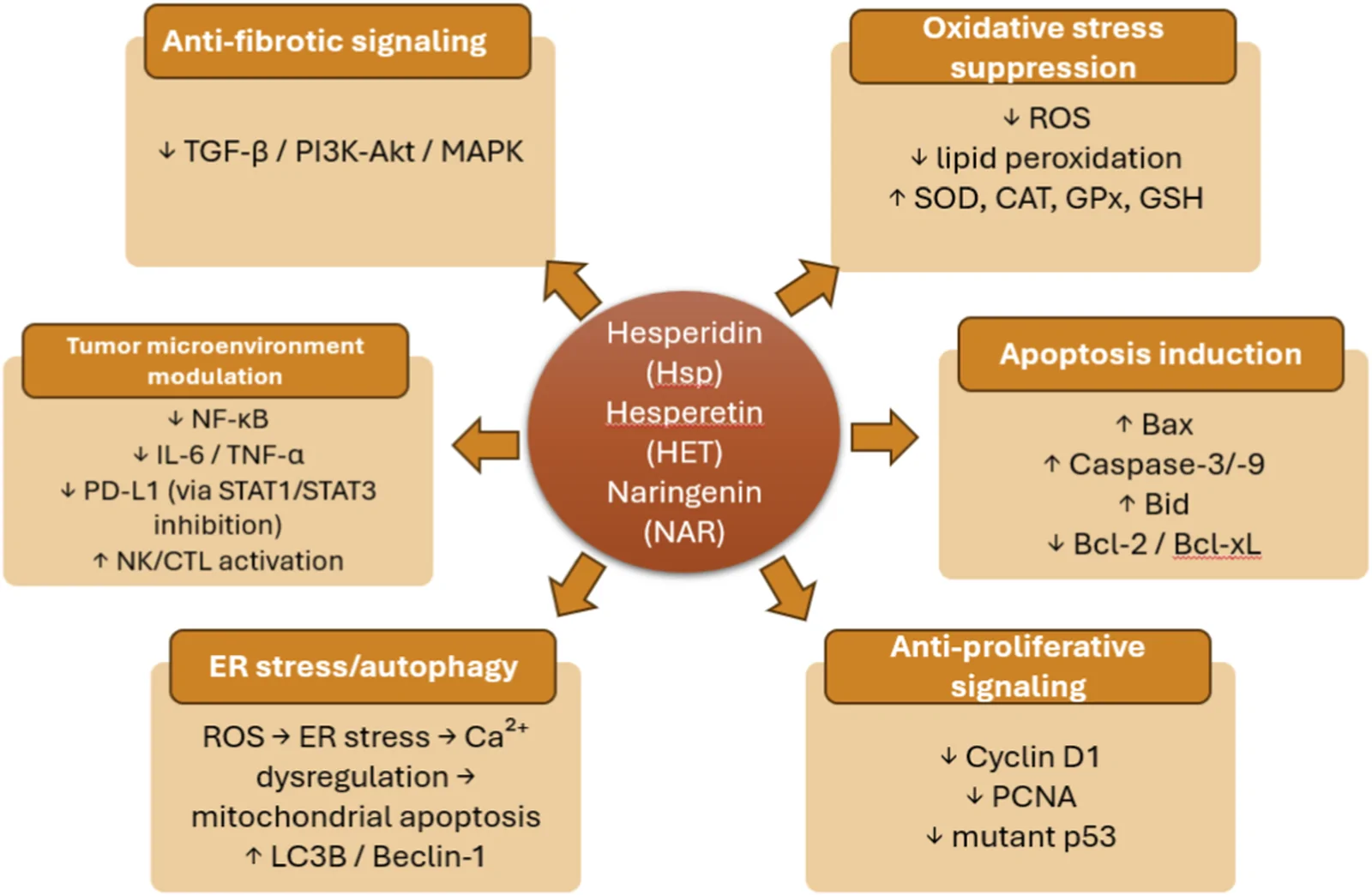
Molecular mechanisms underlying the chemopreventive effects of citrus-derived flavonoids in oral and gastric carcinogenesis. Hesperidin (Hsp), hesperetin (HET), and naringenin (NAR) have been associated with modulation of multiple cellular and molecular processes involved in oxidative stress, proliferation, apoptosis, autophagy, fibrosis, and tumor microenvironment regulation. These flavonoids are reported to reduce oxidative stress in experimental models by lowering ROS and lipid peroxidation, while supporting antioxidant systems such as SOD, CAT, GPx, and GSH. Anti-proliferative effects include changes in expression of proliferation-related markers such as Cyclin D1, PCNA, and mutant p53. Pro-apoptotic signaling is associated with modulation of Bax, Bid, caspases, and inhibition of Bcl-2/Bcl-xL. They may also induce ER stress–related autophagy and mitochondrial apoptosis via ROS-associated Ca^2+^ imbalance and modulation of LC3B and Beclin-1. Additional effects include modulation of TGF-β, PI3K-Akt, and MAPK pathways in fibrosis-related processes, as well as regulation of NF-κB, IL-6, TNF-α, PD-L1, NK cells, and CTLs within the tumor microenvironment. Overall, these findings indicate that citrus-derived flavonoids may contribute to the modulation of biological processes relevant to oral and gastric carcinogenesis in preclinical models.

The DMBA-induced hamster buccal pouch model remains one of the most widely used preclinical models for investigating oral chemoprevention because it recapitulates several histopathological stages of human oral carcinogenesis, ranging from epithelial hyperplasia and dysplasia to squamous cell carcinoma. Evidence derived from this model consistently suggests that citrus flavanones, particularly NAR and HET, modulate multiple early events involved in oral carcinogenesis, including redox imbalance, epithelial proliferation, and tissue remodeling. A major and reproducible finding across studies is the central role of redox regulation in the biological effects associated with chemopreventive potential of these flavanones. DMBA exposure is consistently associated with enhanced lipid peroxidation, impaired antioxidant defenses, and accumulation of oxidative damage, whereas administration of NAR or HET generally restores enzymatic and non-enzymatic antioxidant systems and attenuates oxidative injury (Babukumar et al., 2020; Babukumar et al., 2017; Sulfikkarali et al., 2013) (Table 1; Figure 1). These findings identify restoration of redox homeostasis as a consistent feature of flavanone-mediated chemoprevention in this model (Babukumar et al., 2020; Babukumar et al., 2017; Sulfikkarali et al., 2013; Hanafi et al., 2012). Another consistent observation is the modulation of biomarkers associated with cell proliferation and apoptosis. Across different experimental settings, treatment with NAR or HET has been associated with normalization of proliferative markers, as well as regulation of apoptosis-related proteins such as p53 and caspases (Babukumar et al., 2020; Babukumar et al., 2017; Sulfikkarali et al., 2013). Histopathological improvements, including reduced dysplasia, hyperplasia, and carcinoma incidence, have frequently accompanied these molecular changes (Gurushankar et al., 2015; Babukumar et al., 2020; Babukumar et al., 2017; Sulfikkarali et al., 2013). Collectively, these molecular and histopathological changes support improved regulation of epithelial homeostasis during oral carcinogenesis (Gurushankar et al., 2015; Babukumar et al., 2020; Babukumar et al., 2017; Sulfikkarali et al., 2013).

**TABLE 1:** Summary of chemopreventive effects and molecular mechanisms of NAR, naringin, and hesperetin in oral and GC models.

Study/Model	Dose/Concentration	IC_50_	Findings	Compound/Intervention	Duration/Treatment schedule	Outcome measures/Biomarkers	References
*In vitro*, SGC-7901 GC cells	Serial concentrations (not specified)	Not reported	↓ proliferation, ↓ migration, ↓ invasion, ↓ AKT phosphorylation, ↓ PCNA, ↓ MMP2/9, ↓ Bcl-2, ↓ survivin; ↑ apoptosis, ↑ Bax, ↑ cleaved caspase-3	NAR	Serial concentrations	Proliferation (PCNA), apoptosis (Bax, cleaved caspase-3, Bcl-2, Survivin), migration/invasion (MMP2, MMP9), AKT phosphorylation	Bao et al. (2016)
*In vivo*, Wistar rats; MNNG-induced gastric carcinoma	200 mg/kg (p.o.)	Not applicable	↓ glycoproteins (hexose, hexosamine, sialic acid, fucose); ↓ oxidative stress; ↑ membrane integrity (restoration)	NAR	5 groups, 20-week treatment	Glycoproteins (hexose, hexosamine, sialic acid, fucose)	Ekambaram et al. (2008a)
*In vitro*, SNU-1 gastric carcinoma cells	0–80 μg/mL (24 h); mechanistic studies: 5–20 μg/mL	∼20 μg/mL	↓ proliferation, ↓ PI3K/AKT signaling, ↓ Bcl-2; ↑ apoptosis, ↑ autophagy, ↑ G0/G1 arrest, ↑ Bax, ↑ cleaved caspase-3	Naringin	Dose-dependent	Cell proliferation (CCK8), cell cycle, apoptosis (cleaved caspase-3, Bax, Bcl-2), PI3K/AKT, autophagy	Xu et al. (2021)
*In vitro*, SGC-7901 GC cells	20–160 µM NAR (48 h); selected dose: 40 µM	Not reported	↓ proliferation, ↓ colony formation, ↓ Akt activation; ↑ apoptosis, ↑ caspase-3, ↑ PARP cleavage, ↑ p53	NAR + ABT-737	Dose-dependent	Growth, colony formation, caspase-3, PARP, Akt, p53	Zhang H et al. (2016)
*In vitro*, AGS GC cells	Not specified	Not reported	↓ β-catenin/Tcf signaling; ↔ β-catenin localization; ↔ DNA-binding activity (no change in downstream binding)	NAR	Concentration-dependent	β-catenin/Tcf signaling; nuclear β-catenin; Tcf-4	Lee et al. (2005)
*In vivo*, Wistar rats; MNNG + S-NaCl	200 mg/kg (p.o.)	Not applicable	↓ oxidative stress markers (H_2_O_2_, lipid peroxidation); ↑ antioxidant enzymes (GSH, GPx, GST, GR, etc.); ↑ detoxification enzyme balance (phase I/II normalization)	NAR	5 groups, 20-week treatment	Oxidative stress markers: H2O2, lipid peroxidation, GSH, SOD, CAT, GPx; Phase I/II enzymes	Ganapathy et al. (2008)
*In vivo*, Wistar rats; MNNG + S-NaCl	MNNG 200 mg/kg + naringenin (dose not specified)	Not applicable	↓ tumor size, ↓ body weight loss; ↓ lipid peroxidation; ↓ ROS; ↓ oxidative stress; ↑ SOD, ↑ CAT, ↑ GPx, ↑ GSH, ↑ vitamin C, ↑ vitamin E; ↑ redox balance restoration	NAR	5 groups, 20-week treatment	Tumor size, weight, lipid peroxidation, enzymatic/non-enzymatic antioxidants	Ekambaram et al. (2008b)
*In vivo*, golden Syrian hamsters; DMBA-induced OSCC	50 mg NAR used for nanoparticle preparation (NAR:Eudragit® E:PVA = 1:10:10, w/w/w)	Not reported	↓ tumor formation (complete prevention), ↓ PCNA, ↓ lipid peroxidation; ↑ antioxidants; ↑ p53 expression normalization; ↑ chemopreventive efficacy vs free naringenin	NARNPs	DMBA 0.5%, 3x/week for 14 weeks; 50 mg/kg/day	Histopathology, lipid peroxidation, antioxidants, PCNA, p53; TEM/DLS characterization	Sulfikkarali et al. (2013)
*In vivo*, golden Syrian hamsters; DMBA-induced OSCC	Naringenin nanoparticles vs free naringenin (dose not specified)	Not applicable	↓ nucleic acids, ↓ phenylalanine, ↓ tryptophan; ↑ phospholipids; ↔ biomolecular balance restored; ↑ antitumor effect (NPs > free NAR)	NARNPs vs free NAR	DMBA 0.5%, 3x/week, 14 weeks	FT-Raman spectroscopy; nucleic acids, phenylalanine, tryptophan, phospholipids	Krishnakumar et al. (2013b)
*In vivo*, DMBA-induced hamster buccal pouch	Naringenin nanoparticles vs free naringenin (dose not specified)	Not applicable	↓ proteins, ↓ nucleic acids; ↑ lipids, ↑ glycogen; ↓ membrane disorder; ↑ structural normalization; ↑ chemopreventive effect (NPs > free NAR)	NARNPs vs free NAR	DMBA 0.5%, 3x/week, 14 weeks	FT-IR spectroscopy; proteins, nucleic acids, lipids, glycogen, lipid order, membrane dynamics	Krishnakumar et al. (2013a)
*In vivo*, golden Syrian hamster buccal pouch; DMBA-induced OSCC	Hesperetin (dose not specified)	Not reported	↓ mutant p53, ↓ cyclin D1; ↓ cell proliferation; ↑ caspase-3, ↑ caspase-9; ↑ apoptosis; reversal of tumor markers toward normal	HET	DMBA 0.5%, 3x/week for 10 weeks; oral treatment	Histology, IHC, qRT-PCR, Western blot; apoptotic/proliferative markers	Babukumar et al. (2020)
*In vivo*, hamster buccal pouch; DMBA-induced SCC	Hesperetin 20 mg/kg b.w	Not reported	↓ lipid peroxidation; ↓ tumor progression; ↑ antioxidant enzymes; ↑ detoxification systems; ↑ apoptosis; ↓ proliferation markers; reversal of SCC phenotype	HET	DMBA 0.5%, 3x/week, 10 weeks; oral 20 mg/kg	Lipid peroxidation, enzymatic/non-enzymatic antioxidants, detoxifying enzymes, apoptotic/proliferative markers	Babukumar et al. (2017)
*In vivo*, hamster buccal pouch; DMBA-induced OSCC	Hesperetin-loaded nanoparticles vs hesperetin (dose not specified)	Not applicable	↓ tumor metabolism; ↓ NADH & FAD fluorescence; ↓ optical redox ratio in tumors; ↑ redox ratio after treatment; ↑ collagen/NADH/FAD normalization; ↑ metabolic balance restoration; nanoparticle form showed stronger effect than free hesperetin	HETNPs	DMBA 0.5%, 3x/week; oral treatment	Tissue autofluorescence spectroscopy; collagen, NADH, FAD; optical redox ratio (FAD/(NADH + FAD))	Gurushankar et al. (2015)
*In vivo*, F344 rats; 4-NQO-induced oral carcinoma	Diosmin 1,000 ppm, hesperidin 1,000 ppm (alone or combination)	Not applicable	↓ tongue carcinoma incidence; ↓ preneoplastic lesions; ↓ cell proliferation (BrdU index, NOR proteins); ↓ polyamines; ↓ hyperplasia/dysplasia; ↓ tumor development; ↑ inhibition of oral carcinogenesis (both initiation and post-initiation stages)	Diosmin & Hsp	Diet containing compounds; initiation/post-initiation; 32-week study	Tumor incidence, polyamine levels, BrdU labeling, nucleolar organizer region proteins	Tanaka et al. (1997)

SGC-7901, human gastric cancer cell line; SNU-1, human gastric cancer cell line; AGS, human gastric adenocarcinoma cell line; DMBA-induced OSCC, 7,12-Dimethylbenz(a)anthracene-induced oral squamous cell carcinoma; MNNG, N-methyl-N′-nitro-N-nitrosoguanidine; S-NaCl, saturated sodium chloride; Naringenin-loaded nanoparticles, NARNPs; Hesperetin-loaded nanoparticles, HETNPs; Naringenin, NAR; Hesperetin, HET; Hsp, Hesperidin; Auraptene, AUR; PCNA, proliferating cell nuclear antigen; MMP2, matrix metalloproteinase-2; MMP9, matrix metalloproteinase-9; Bax, Bcl-2-associated X protein; Bcl-2, B-cell lymphoma 2; PI3K/AKT, phosphoinositide 3-kinase/protein kinase B pathway; PARP, poly(ADP-ribose) polymerase; GSH, reduced glutathione; SOD, superoxide dismutase; CAT, catalase; GPx, glutathione peroxidase; NADH, nicotinamide adenine dinucleotide; FAD, flavin adenine dinucleotide; LPO, lipid peroxidation; HBP, hamster buccal pouch; qRT-PCR, quantitative real-time polymerase chain reaction; IHC, immunohistochemistry; SCC, squamous cell carcinoma.

↑ increase/enhancement/restoration; ↓ decrease/inhibition; ↔ normalization/balance restored.

Despite these overall consistencies, several limitations and discrepancies remain. Most available studies have been conducted in a single animal model using relatively short intervention periods and heterogeneous dosing regimens, making direct comparison among studies difficult. Furthermore, molecular endpoints vary substantially across investigations, and few studies have systematically evaluated dose-response relationships or long-term preventive efficacy. Consequently, although modulation of oxidative stress and proliferation-related pathways appears reproducible, the relative contribution of individual signaling pathways to the observed biological effects in this model system remains insufficiently defined. An emerging theme in this field is the potential advantage of nanochemopreventive formulations. Poor aqueous solubility, extensive metabolism, and limited bioavailability are recognized constraints for the clinical translation of flavanones (Sulfikkarali et al., 2013). Studies employing nanoparticle-based formulations generally report greater biological efficacy than native flavanones, including improved molecular, biochemical, and histopathological outcomes (Gurushankar et al., 2015; Sulfikkarali et al., 2013; Krishnakumar et al., 2013a; Krishnakumar et al., 2013b). Spectroscopic analyses, including FT-Raman, FT-IR, and autofluorescence approaches, further suggest that nanoformulations may better preserve biomolecular integrity and cellular metabolic status (Gurushankar et al., 2015; Krishnakumar et al., 2013a; Krishnakumar et al., 2013b). Nevertheless, these findings are currently derived from a limited number of preclinical studies, and standardized comparisons between free and nanoencapsulated flavanones are still lacking (Gurushankar et al., 2015; Sulfikkarali et al., 2013; Krishnakumar et al., 2013a; Krishnakumar et al., 2013b). Beyond direct effects on epithelial cells, evidence also indicates that citrus flavanones may modulate the TME. For example, NAR has demonstrated antifibrotic activity in human gingival fibroblasts through regulation of transforming growth factor-beta-, phosphoinositide 3-kinase–protein kinase B (PI3K/AKT)-, and mitogen-activated protein kinase (MAPK)-related signaling, suggesting a potential role in preventing microenvironmental alterations associated with oral submucous fibrosis and malignant progression (Samyuktha Aarthi et al., 2025). In addition, combination strategies involving flavanones and other dietary phytochemicals, such as curcumin, Hsp, or auraptene, have shown additive or potentially synergistic preventive effects in experimental oral carcinogenesis models (Samyuktha Aarthi et al., 2025; Tanaka et al., 1998). These findings support the concept that multi-target interventions may provide greater biological modulation of carcinogenesis-related processes than single-agent approaches (Samyuktha Aarthi et al., 2025; Tanaka et al., 1998; Tanaka et al., 1994). Overall, available preclinical evidence supports the chemopreventive potential of NAR and HET in oral carcinogenesis, although clinical validation, dose optimization, and standardized comparative studies remain essential before translation into practice.

### 5.2 Chemopreventive insights of NAR and naringin in GC

GC remains a major cause of cancer-related morbidity and mortality worldwide, emphasizing the need for effective preventive strategies capable of interrupting early carcinogenic events and reducing disease progression (Machlowska et al., 2020). Among dietary phytochemicals, citrus flavanones have attracted attention because they target multiple processes involved in gastric carcinogenesis; however, available evidence should be interpreted separately for chemopreventive animal models and therapeutic studies using established GC cell lines (Ganapathy et al., 2008; Bao et al., 2016; Xu et al., 2021). Most *in vitro* studies performed in established GC cell lines primarily evaluate therapeutic responses in transformed cancer cells, whereas chemically induced animal models more closely reflect chemopreventive interventions during tumor initiation and promotion. Evidence from GC cell lines, including SGC-7901, SNU-1, and AGS cells, consistently demonstrates that NAR and naringin suppress malignant cellular phenotypes, suggesting antitumor activity in established cancer cells rather than direct chemopreventive effects (Bao et al., 2016; Xu et al., 2021; Lee et al., 2005; Zhang H. et al., 2016). Across these models, flavanone treatment reduced cellular proliferation, migration, and invasion while promoting programmed cell death (Bao et al., 2016; Xu et al., 2021; Lee et al., 2005; Zhang H. et al., 2016). A recurrent finding is the inhibition of PI3K/AKT signaling, accompanied by reduced expression of proliferative and survival-associated proteins, including proliferating cell nuclear antigen, B-cell lymphoma 2 (Bcl-2), and survivin, together with increased expression of Bax and cleaved caspase-3 (Bao et al., 2016; Xu et al., 2021; Zhang H. et al., 2016). In addition, suppression of matrix metalloproteinase-2 (MMP-2) and MMP9 expression was consistently associated with decreased invasive capacity, whereas inhibition of β-catenin/Tcf transcriptional activity suggested interference with oncogenic transcriptional programs involved in tumor progression (Bao et al., 2016; Lee et al., 2005). Naringin was also reported to induce G0/G1 arrest and activate autophagy in SNU-1 cells, and pharmacological inhibition of autophagy partially attenuated apoptosis, indicating functional crosstalk between these processes (Xu et al., 2021). Collectively, these studies demonstrate broad suppression of signaling pathways governing GC cell survival, invasion, and stress adaptation (Bao et al., 2016; Xu et al., 2021; Lee et al., 2005; Zhang H. et al., 2016).

Across chemically induced models, NAR consistently restored redox homeostasis, enhanced endogenous antioxidant defenses, and normalized carcinogen-detoxifying enzyme systems, changes that were accompanied by preservation of gastric tissue architecture and reduced tumor development (Ganapathy et al., 2008; Ekambaram et al., 2008a; Ekambaram et al., 2008b). Preservation of glycoprotein homeostasis and improvement of histopathological features further suggest that NAR may maintain epithelial integrity and suppress progression from preneoplastic lesions to overt malignancy (Ekambaram et al., 2008a; Ekambaram et al., 2008b). An important observation emerging from animal studies is that the timing of intervention substantially influences efficacy. Preventive administration of NAR before or during carcinogen exposure generally produced greater protective effects than post-initiation treatment, supporting the concept that modulation of oxidative stress and detoxification pathways may be particularly relevant during early stages of gastric carcinogenesis (Ganapathy et al., 2008). However, direct comparisons among studies remain challenging because of heterogeneity in experimental protocols, treatment duration, and administered doses. In addition, long-term safety, optimal dosing regimens, and pharmacokinetic-pharmacodynamic relationships remain insufficiently characterized.

**FIGURE 2:**
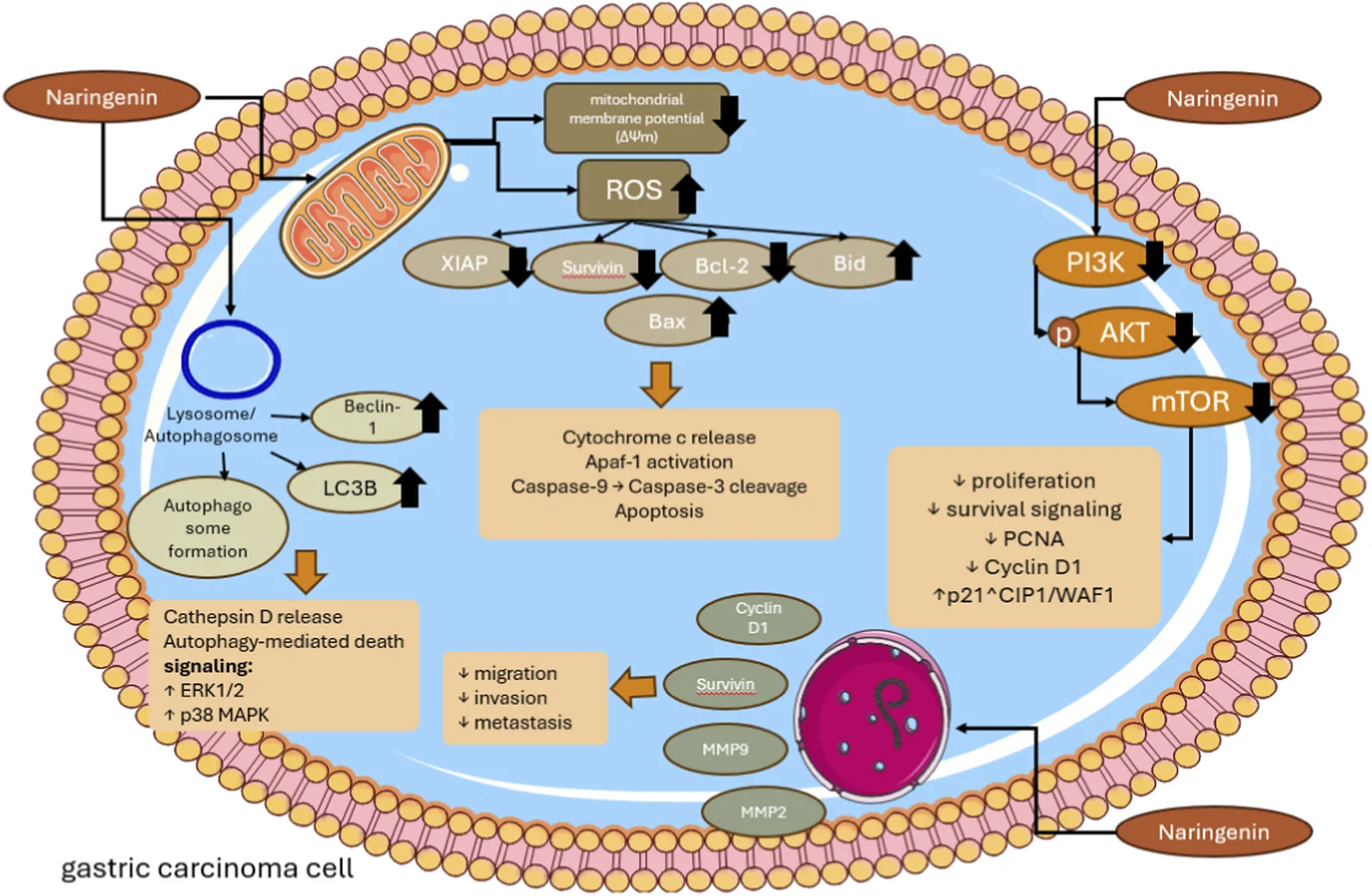
Intracellular chemopreventive mechanisms of naringenin and naringin in gastric carcinoma cells. Naringenin (NAR) and naringin may modulate gastric carcinogenesis through regulation of oxidative stress, proliferation, apoptosis, autophagy, and metastatic signaling pathways. These flavonoids have been reported to be associated with inhibition of PI3K/Akt/mTOR and β-catenin/Tcf signaling and reduced expression of PCNA, Cyclin D1, MMP2, and MMP9. Pro-apoptotic effects appear to involve Bax, Bid, cytochrome c, and caspase-3/-9 activation, along with decreased Bcl-2 and survivin levels. Naringin has also been linked to lysosomal-autophagic processes via LC3B, Beclin-1, and Cathepsin D. Antioxidant effects include reported restoration of SOD, CAT, GPx, and GSH with concurrent reduction of ROS and lipid peroxidation. *In vivo* studies suggest potential attenuation of gastric lesions and tumor burden in chemically induced models, while nanoparticle formulations may enhance delivery and biological activity.

Comparable chemopreventive effects have also been reported for other citrus flavonoids, including Hsp, diosmin, and auraptene, suggesting that modulation of oxidative stress, epithelial proliferation, and carcinogen detoxification represents a shared protective mechanism across this compound class (Ganapathy et al., 2008; Ekambaram et al., 2008a; Ekambaram et al., 2008b; Tanaka et al., 1997). These findings collectively suggest that modulation of oxidative stress, epithelial proliferation, and carcinogen detoxification may represent shared biological processes associated with chemopreventive potential across citrus flavonoids. Epidemiological investigations further support a protective association between flavonoid-rich diets and GC risk. Pooled analyses and population-based studies have reported inverse associations between dietary intake of citrus fruits, flavanones, flavanols, and total flavonoids and the risk of GC, although the magnitude of these associations varies among populations and study designs (Bertuccio et al., 2019; Vitelli Storelli et al., 2019; Vitelli-Storelli et al., 2020). However, these observational studies cannot establish causality because dietary, genetic, and lifestyle factors may confound the reported associations. Overall, experimental evidence supports the chemopreventive potential of NAR and naringin in gastric carcinogenesis, although translation to clinical prevention remains limited by the predominance of preclinical studies and the lack of standardized human investigations (Ganapathy et al., 2008; Bao et al., 2016; Xu et al., 2021; Lee et al., 2005; Zhang H. et al., 2016; Ekambaram et al., 2008a; Ekambaram et al., 2008b; Tanaka et al., 1997; Bertuccio et al., 2019; Vitelli Storelli et al., 2019; Vitelli-Storelli et al., 2020) (Figure 2).

## 6 Mechanistic insights into oral and GC cell death pathways mediated by NAR, naringin, and HET

Programmed cell death in oral and GC is regulated by interconnected stress-response and survival pathways, including oxidative stress, mitochondrial dysfunction, endoplasmic reticulum (ER) stress, autophagy, and PI3K/Akt/mechanistic target of rapamycin (mTOR) signaling (Zhao, 2025; Hu et al., 2023). Experimental studies indicate that citrus flavanones, particularly NAR, naringin, and HET, can modulate several of these pathways. However, it should be noted that most available evidence has been generated in established cancer cell lines and therefore primarily reflects antitumor or chemotherapeutic activity rather than direct chemopreventive effects (Table 2).

**TABLE 2:** Summary of core effects and stress-death-related molecular mechanisms of NAR, naringin, and HET in oral and GC models.

Compound	Model	Dose/Concentration	IC_50_	Findings	Mechanism/pathways modulated	Key molecular markers/readouts	References
NAR	Human OSCC cells (*in vitro*)	Not specified	Not reported	↑ ROS production; ↑ ER stress; ↑ Ca^2+^ dysregulation; ↑ caspase cascade; ↑ mitochondrial dysfunction; ↑ apoptosis; ↑ autophagy switched to pro-death	ER stress-mediated apoptosis; autophagy modulation; ROS	Caspase cascade, mitochondrial dysfunction, intracellular ROS, Ca^2+^	Liu et al. (2022)
NAR	CAL-27 tongue carcinoma cells (*in vitro*)	Not specified	Not reported	↑ apoptosis; ↑ ROS; ↑ Bid; ↓ Bcl-xl; ↑ mitochondrial apoptotic signaling	ROS-mediated apoptosis; Bid/Bcl-xL signaling	ROS, Bid upregulation, Bcl-xL downregulation, JC-1, Annexin V-FITC	Du et al. (2024)
NAR + Cisplatin	TSCC (HNO-97) + normal oral epithelial cells	NAR + Cisplatin Different concentrations (not specified); 48 h treatment	Determined by MTT assay (value not reported in provided section)	↑ apoptosis; ↑ caspase-3; ↑ cytotoxicity; ↓ cell viability; ↓ Bax (unexpected report); ↓ IC50 with combination vs single drugs	Caspase-dependent apoptosis; enhanced cytotoxicity	Caspase-3, Bax, Bcl-2, IC50, MTT	Atef et al. (2025)
Hsp	KB oral cancer cells (*in vitro*)	Various concentrations (not specified); 48 h treatment	89.17 µM	↓ proliferation; ↓ TNF-α, ↓ IL-1β, ↓ IL-6, ↓ NF-κB, ↓ Bcl-2; ↑ apoptosis; ↑ Bax expression	Apoptosis and inflammatory signaling modulation	TNF-α, IL-1β, IL-6, NF-κB, Bcl-2, BAX mRNA	Jayaraman et al. (2024)
NAR -BSA nanoparticles (NGN-BSA)	Gastric cancer rats + NGN-BSA nanoparticles (*in vivo* + *in vitro*)	Nanoparticles (dose not specified)	Not applicable	↓ ROS; ↓ TNF-α; ↓ proliferation; ↓ invasion; ↓ gastric ulcer; ↑ apoptosis; ↑ cell death; ↑ anti-tumor activity of nanoparticle formulation	ROS modulation; TNF-α downregulation; oxidative & inflammatory stress	Intracellular ROS, TNF-α mRNA, MTT proliferation, Transwell migration, apoptosis	Liu et al. (2024)
Naringin	AGS gastric cancer cells (*in vitro*)	Not specified	Not reported	↓ PI3K/Akt/mTOR signaling; ↑ lysosomal membrane permeabilization (LMP); ↑ autophagy; ↑ ROS; ↑ ERK1/2 & p38 MAPK; ↑ Cathepsin D release; ↑ apoptosis via lysosomal + mitochondrial pathways	Lysosomal/autophagy-mediated cell death; ROS-mediated stress; PI3K/Akt/mTOR inhibition; MAPK activation	LC3B, Beclin-1, LAMP1, Cathepsin D, Bad, Bcl-xL, ERK1/2, p38 MAPK	Raha et al. (2020)
NAR + ABT-263	SGC-7901 gastric cancer cells (*in vitro*)	Nar + ABT-263 (combo)	Not reported	↓ p-AKT; ↓ proliferation; ↑ apoptosis; ↑ caspase activation; ↑ synergy vs single agents; AKT total protein ↔ unchanged	PI3K/Akt pathway inhibition; Bcl-2 inhibition; apoptosis	p-AKT, total AKT, caspase-3, apoptosis rate	Ye et al. (2019)
Naringin	AGS gastric cancer cells (*in* *vitro*)	EC_50_/IC_50_ not explicitly reported; effective concentration (EC) = 2 mM (50% growth inhibition observed at 3 mM within 24 h)	Not reported	↓ PI3K/Akt/mTOR cascade; ↑ autophagy; ↑ MAPK activation; ↑ LC3B, Beclin-1; ↑ p21; ↓ proliferation; apoptosis ↔ not dominant (autophagic death primary)	Autophagy-mediated growth inhibition; PI3K/Akt/mTOR suppression; MAPK activation	Beclin-1, LC3B, cytoplasmic vacuoles, autophagosomes, p21^CIPI/WAFI	Raha et al. (2015)
HET	Gastric cancer cells + xenograft mice (*in vivo* + *in vitro*)	Hesperetin (dose not specified)	Not reported	↑ ROS; ↑ apoptosis; ↓ mitochondrial membrane potential; ↑ caspase-3/9, Cyt C, Apaf-1, Bax; ↓ Bcl-2; ↓ tumor growth *in vivo*	Mitochondrial apoptosis; ROS-mediated stress; intrinsic apoptotic pathway	ROS, Δψm, AIF, Apaf-1, Cyt C, caspase-3/9, Bax, Bcl-2	Zhang et al. (2015)
HET	AGS gastric cancer cells (*in vitro*)	0, 3.13, 6.25, 12.5, 25, 50, 100, 200 μM (24 h treatment); mechanistic studies used 6.25–50 μM	Not reported (effective doses: 6.25–50 μM)	↑ apoptosis; ↑ caspase-3/7/8/9; ↓ XIAP, ↓ survivin; ↓ PI3K/Akt; ↓ FAK; ↑ AMPK activation; ↓ mTOR signaling; ↑ mitochondrial apoptosis	Caspase-mediated apoptosis; PI3K/AKT/mTOR/STAT3 inhibition	Caspase-3/7/8/9, XIAP, survivin, Bcl-2/Bax ratio, FAK, PI3K, AKT, AMPK, mTOR, STAT3, c-Myc	Yang et al. (2025)
HET + Daunorubicin (HE/DA/MPEG-PCL)	Gastric cancer cells (*in vitro* nanoparticle system)	HE/DA/M nano-micelles: 0, 20, 40, 60, 80, and 100 ng/mL (24 h)	Not reported	↑ apoptosis; ↑ cytotoxicity; ↑ tumor targeting; ↓ proliferation; ↑ drug accumulation; ↓ off-target toxicity; ↑ synergistic anticancer effect	Mitochondrial apoptosis; enhanced cytotoxicity via targeted nanoparticle delivery	Particle size (∼38 nm), zeta potential, apoptosis, proliferation, cytotoxicity	Zhen et al. (2017)

ROS, reactive oxygen species; Δψm, mitochondrial membrane potential; Caspase-3/7/8/9, cysteine-aspartic proteases; BAX, Bcl-2-associated X protein; Bcl-2, B-cell lymphoma 2; Bid, BH3 interacting-domain death agonist; LC3B, microtubule-associated protein 1A/1B-light chain 3; Beclin-1, autophagy-related protein; LAMP1, lysosomal-associated membrane protein 1; Cathepsin D, lysosomal protease; p-AKT, phosphorylated protein kinase B; Total AKT, total protein kinase B; p21^CIPI/WAFI, cyclin-dependent kinase inhibitor; ERK1/2, extracellular signal-regulated kinase 1/2; p38 MAPK, mitogen-activated protein kinase; TNF-α, tumor necrosis factor alpha; IL-1β, interleukin-1 beta; IL-6, interleukin-6; NF-κB, nuclear factor kappa-light-chain-enhancer of activated B cells; TNF-α mRNA, tumor necrosis factor alpha messenger RNA; Cyt C, cytochrome c; AIF, apoptosis-inducing factor; Apaf-1, apoptotic protease activating factor-1; Δψm disruption, loss of mitochondrial membrane potential; Annexin V-FITC, fluorescein-labeled annexin V; IC50, half-maximal inhibitory concentration; MTT, 3-(4,5-dimethylthiazol-2-yl)-2,5-diphenyltetrazolium bromide; Particle size, hydrodynamic diameter of nanoparticles; Zeta potential, nanoparticle surface charge; ROS agonist, exogenous reactive oxygen species enhancer; HETNP, hesperetin-loaded nanoparticles; NGN-BSA, naringenin–bovine serum albumin nanoparticles; HE/DA/MPEG-PCL, hesperetin/daunorubicin loaded nanoparticles with MPEG-PCL carrier; PD-L1, programmed death-ligand 1; STAT1/3, signal transducer and activator of transcription 1/3; EMT, epithelial-mesenchymal transition.

↑ increase/enhancement/restoration; ↓ decrease/inhibition; ↔ normalization/balance restored.

### 6.1 Stress-induced cell death pathways in OSCC modulated by flavonoids

A consistent finding across OSCC models is that citrus flavanones disrupt redox homeostasis, triggering interconnected stress responses involving mitochondrial dysfunction, ER stress, and programmed cell death (Jayaraman et al., 2024; Atef et al., 2025; Du et al., 2024; Liu et al., 2022; Li et al., 2023; Dewenter et al., 2023). Among citrus flavanones, NAR has been most extensively investigated. Studies in OSCC cell lines demonstrated that NAR treatment increased intracellular ROS accumulation, promoted mitochondrial membrane depolarization, activated caspase-dependent apoptosis, and altered the balance between pro- and anti-apoptotic proteins, including increased Bid or Bax expression together with reduced Bcl-xL or Bcl-2 levels (Du et al., 2024; Liu et al., 2022). In parallel, NAR was reported to modulate autophagic responses through ROS-dependent ER stress signaling, suggesting functional crosstalk between autophagy and apoptosis under conditions of sustained cellular stress (Liu et al., 2022). Current evidence suggests that sustained flavanone-induced autophagy may contribute to, rather than prevent, cell death in OSCC cells (Liu et al., 2022). Another recurrent observation is the interaction between apoptotic signaling and inflammatory pathways. Hsp reduced proliferation and enhanced apoptosis in KB oral cancer cells while simultaneously suppressing inflammatory mediators, including TNF-α, IL-1β, IL-6, and NF-κB signaling (Jayaraman et al., 2024). These observations indicate that suppression of pro-survival inflammatory signaling may reinforce flavonoid-induced apoptosis, although the relative contribution of inflammatory pathways remains incompletely defined (Jayaraman et al., 2024).

Several studies have also explored flavonoids as adjuvants to conventional chemotherapy. NAR enhanced cisplatin-induced cytotoxicity in HNO-97 tongue squamous carcinoma cells through activation of caspase-3-dependent apoptosis and modulation of mitochondrial apoptotic regulators, including Bcl-2 and Bax (Atef et al., 2025). Similarly, combined treatment with Hsp and cisplatin increased oxidative stress, disrupted mitochondrial function, and enhanced cytotoxicity in Hep-2 laryngeal carcinoma cells, suggesting potential chemosensitizing effects (Çınar et al., 2026). Because these studies were performed in established cancer cell lines, they primarily support therapeutic combination strategies rather than chemopreventive activity (Atef et al., 2025; Çınar et al., 2026). Supportive evidence from other phytochemicals further reinforces the importance of oxidative stress-mediated cell death in oral malignancies. Supportive evidence from related phytochemicals also underscores the importance of ROS-dependent stress responses in head and neck malignancies. For example, naringin induced ROS-mediated ER stress, cell-cycle arrest, and apoptosis in nasopharyngeal carcinoma cells (Chen et al., 2024). Collectively, available evidence supports a coordinated mechanism in which citrus flavanones induce OSCC cell death through interconnected regulation of oxidative stress, mitochondrial dysfunction, ER stress, autophagy, and apoptosis (Jayaraman et al., 2024; Atef et al., 2025; Du et al., 2024; Liu et al., 2022; Çınar et al., 2026; Chen et al., 2024; Tan et al., 2025; Chang et al., 2017). Nevertheless, mechanistic validation in animal models and clinical settings remains limited, and the relative contribution of these pathways to chemopreventive versus therapeutic effects requires further investigation.

### 6.2 Cell death pathways in GC: mitochondrial, autophagic, and PI3K/Akt/mTOR signaling

Cell survival and death in GC are regulated by interconnected signaling networks involving mitochondrial apoptosis, lysosomal membrane permeabilization (LMP), autophagy, and PI3K/Akt/mTOR signaling (Bravo et al., 2019; Rong et al., 2020). Accumulating evidence indicates that citrus flavonoids can modulate several of these pathways. However, because most available studies have been performed in established GC cell lines, these findings should primarily be interpreted as evidence of antitumor or chemotherapeutic activity rather than direct chemopreventive effects. One of the most consistent findings across GC models is that oxidative stress serves as an upstream regulator of flavonoid-induced growth inhibition (Liu et al., 2024; Raha et al., 2020). Compared with free NAR, NAR nanoparticles (NGN-BSA) improved cellular uptake and enhanced antitumor efficacy, as reflected by reduced oxidative and inflammatory markers, inhibition of proliferation and migration, and improved gastric histopathology, supporting nanoformulation as a strategy to overcome poor bioavailability (Liu et al., 2024).

Another recurrent finding is the prominent role of lysosomal and autophagy-associated pathways in naringin-treated GC cells. Studies in AGS and other GC cell lines demonstrated increased autophagosome formation, activation of autophagy-related proteins such as LC3B and Beclin-1, and induction of LMP accompanied by cytosolic release of cathepsin D (Raha et al., 2020; Raha et al., 2015). These events were associated with suppression of PI3K/Akt/mTOR signaling and activation of stress-responsive MAPK pathways, including ERK1/2 and p38 MAPK (Raha et al., 2020; Raha et al., 2015). Pharmacological studies indicate that ROS generation and lysosomal dysfunction act upstream of autophagy, supporting a coordinated mechanism in which oxidative stress suppresses GC cell growth through interconnected lysosomal, autophagic, and PI3K/Akt/mTOR signaling pathways, although the relative contribution of autophagy versus apoptosis remains unresolved (Raha et al., 2020; Raha et al., 2015). Mitochondrial apoptosis also appears to represent an important target of citrus flavonoids in GC cells. HET consistently induced mitochondrial dysfunction, increased intracellular ROS generation, and disrupted mitochondrial membrane potential, leading to activation of intrinsic apoptotic pathways (Yang et al., 2025; Zhang et al., 2015). Across different experimental models, HET treatment increased the expression of pro-apoptotic mediators, including Bax, Apaf-1, cytochrome c, and caspases, while reducing the expression of anti-apoptotic proteins such as Bcl-2, survivin, and XIAP (Yang et al., 2025; Zhang et al., 2015). In addition, HET inhibited multiple pro-survival signaling pathways, including PI3K, AKT, FAK, mTOR, and STAT3/c-Myc signaling, while enhancing AMPK activity, suggesting simultaneous targeting of several survival networks (Yang et al., 2025). Collectively, these findings identify mitochondrial dysfunction and coordinated inhibition of multiple survival pathways as central mechanisms underlying HET-induced antitumor activity in GC cells (Yang et al., 2025; Zhang et al., 2015).

Several investigations have further explored combinatorial approaches designed to enhance flavonoid efficacy. Co-treatment of SGC7901 cells with NAR and the Bcl-2 inhibitor ABT-263 produced greater antiproliferative and pro-apoptotic effects than either agent alone, accompanied by reduced AKT phosphorylation and enhanced activation of apoptotic pathways (Ye et al., 2019). Similarly, nanoparticle-mediated co-delivery of HET and daunorubicin improved tumor-targeted accumulation and enhanced cytotoxic responses while reducing systemic toxicity in preclinical models (Zhen et al., 2017). These observations support the potential of flavanones as adjuvants to conventional chemotherapy, although clinical validation remains limited. Consistent with these findings, flavonoids isolated from *Citrus aurantium* also inhibited GC cell proliferation and promoted cell-cycle arrest and programmed cell death, supporting the notion that coordinated regulation of proliferative and survival pathways is a shared feature of citrus flavonoids (Lee et al., 2012). Overall, available evidence supports a coordinated mechanism whereby citrus flavonoids suppress GC progression through integrated regulation of oxidative stress, lysosomal-autophagic signaling, mitochondrial apoptosis, and PI3K/Akt/mTOR-dependent survival pathways (Liu et al., 2024; Raha et al., 2020; Raha et al., 2015; Yang et al., 2025; Zhang et al., 2015; Ye et al., 2019; Zhen et al., 2017; Lee et al., 2012). However, translation of these findings remains constrained by the predominance of *in vitro* studies and the limited availability of standardized animal and clinical investigations.

## 7 Citrus flavanones in TME regulation: immune, angiogenic, and nanodelivery effects

TME plays a central role in cancer progression by regulating immune surveillance, inflammatory signaling, angiogenesis, and metastatic dissemination (Hinshaw and Shevde, 2019). Increasing evidence suggests that citrus flavonoids, including NAR, HET, and Hsp, may modulate several components of the TME. Increasing evidence suggests that citrus flavonoids, including NAR, HET, and Hsp, modulate multiple components of the TME, although current evidence remains largely preclinical. NAR has been reported to enhance antitumor immune responses by promoting activation of CD169-positive macrophages, thereby increasing IL-12 and CXCL10 production and facilitating CTL recruitment in murine OSCC models (Kawaguchi et al., 2022; Liu and Jiao, 2025). In addition, NAR promoted dendritic cell differentiation and maturation through the FKBP4/NR3C1/nuclear factor erythroid 2–related factor 2 axis, potentially strengthening antigen presentation and sustaining NK cell and CTL activity (Xiong et al., 2021). Complementary studies further suggest that modulation of immune-regulatory pathways may enhance NK-cell differentiation and cytotoxic function (Lian et al., 2018). Collectively, these findings support coordinated activation of innate and adaptive antitumor immunity by citrus flavonoids in preclinical models (Kawaguchi et al., 2022; Xiong et al., 2021; Lian et al., 2018). Immune evasion through checkpoint signaling represents another important feature of the TME. Experimental evidence in OSCC models demonstrated that Hsp attenuated IFN-γ-induced PD-L1 expression by suppressing STAT1 and STAT3 phosphorylation, thereby reducing tumor cell proliferation, migration, and invasiveness (Wudtiwai et al., 2021). These findings suggest that flavonoids may partially overcome tumor-associated immune suppression, although evidence for enhanced checkpoint inhibitor responses remains limited (Wudtiwai et al., 2021). Inflammatory cytokine signaling is also closely linked to tumor progression and epithelial-mesenchymal transition (EMT). Flavonoids also suppress inflammatory signaling associated with EMT by inhibiting IL-6/IL-8–STAT3 and NF-κB pathways while restoring epithelial characteristics and reducing invasive phenotypes (Wudtiwai et al., 2021; Hsu et al., 2021; Maquera-Huacho et al., 2023). Another consistent feature of citrus flavonoids is their antiangiogenic activity. Experimental studies demonstrate reduced VEGF and angiopoietin-1 expression together with suppression of inflammatory pathways linked to neovascularization, collectively limiting vascular remodeling and tumor progression (Maquera-Huacho et al., 2023; Gurushankar et al., 2014a; Hana and Bawi, 2018; Echizen et al., 2018; Gurushankar et al., 2014b).

Nanoformulated HET exhibited improved aqueous solubility, cellular uptake, and sustained release, resulting in enhanced antitumor efficacy compared with native HET (Gurushankar et al., 2014b). These findings are consistent with observations in other sections indicating that nanoencapsulation may substantially improve flavonoid efficacy by enhancing tissue targeting and intracellular accumulation. Nevertheless, standardized comparisons between native and nanoformulated flavonoids remain limited, and further pharmacokinetic and safety studies are needed before clinical translation can be considered (Gurushankar et al., 2014b). Emerging evidence further suggests that interactions among the microbiota, stromal cells, and inflammatory mediators may influence gastrointestinal carcinogenesis (Gall et al., 2015; You et al., 2024; Lei et al., 2024; Kim et al., 2021; Zhang Q. et al., 2016). *Helicobacter* pylori-induced stromal activation promotes inflammatory cytokine production and EMT, thereby contributing to gastric tumor progression (Zhang Q. et al., 2016). Interestingly, HET has demonstrated antibacterial activity against *H. pylori* and reduced the expression of several bacterial virulence-associated genes, suggesting that flavonoids may indirectly modulate tumor-promoting microenvironmental signals by influencing microbial communities (Kim et al., 2021). Alterations in oral and upper gastrointestinal microbiota have also been associated with gastric carcinogenesis, although their functional relevance remains incompletely understood (Gall et al., 2015; You et al., 2024; Lei et al., 2024). Consequently, modulation of host-microbiota interactions represents an emerging area that warrants further investigation in the context of flavonoid-based cancer prevention. Overall, preclinical evidence indicates that citrus flavonoids remodel multiple components of the TME including antitumor immunity, inflammatory signaling, angiogenesis, and host, microbiota interactions. However, mechanistic integration and clinical validation remain limited, highlighting the need for standardized *in vivo* models and translational studies to define the contribution of TME modulation to oral and gastric cancer prevention and therapy.

## 8 Flavanone regulation of tumor progression: epigenetic and non-coding RNA (ncRNA) pathways

Tumor progression is driven by coordinated alterations in cell-cycle regulation, EMT, invasion, metastatic dissemination, stemness, and intercellular communication (Micalizzi et al., 2010). Increasing evidence indicates that citrus flavanones, particularly NAR, naringin, and HET, regulate tumor progression through epigenetic mechanisms, ncRNA networks, and signaling pathways controlling proliferation and metastasis (Babukumar et al., 2018; Liang ZF. et al., 2025; Tajaldini et al., 2020; Zhu et al., 2023; Maggioni et al., 2014; Wang SW. et al., 2021). However, because the majority of available evidence originates from *in vitro* studies and xenograft models, these observations should be interpreted primarily as evidence of antitumor activity, whereas their direct contribution to cancer chemoprevention remains to be established (Table 3).

**TABLE 3:** HET, NAR, and naringin as core flavanone interventions modulating tumor progression: Epigenetic, cytostatic, and exosomal mechanisms.

Model	Dose/Concentration	IC_50_	Findings	Mechanism/Pathways	Key molecular markers/readouts	References
Gastric cancer cells + nude mice (*in vitro* + *in* *vivo*)	Hesperetin (dose not specified)	Not reported	↓ DOT1L protein stability; ↓ H3K79 methylation; ↓ migration; ↓ invasion; ↓ metastasis-related genes; ↑ epigenetic tumor suppression via CBP regulation	Epigenetic modulation via DOT1L degradation; reduced H3K79 methylation; transcriptional repression of metastasis-related genes	DOT1L, H3K79me, CBP, migration/invasion assays, qPCR, ChIP-qPCR, Western blot	Wang S. W. et al. (2021)
SCC-25 oral cancer cells (*in vitro*)	Naringenin (MYR also tested)	Not reported	↓ proliferation; ↓ migration; ↓ Cyclin D1; ↓ Cyclin B1 (cell cycle arrest); ↔ apoptosis (not primary mechanism); ↓ cell motility	Cytostatic effect via cell cycle arrest (G0/G1 and G2/M); inhibition of proliferation and motility	Cyclin D1, Cyclin B1, wound-healing assay, transwell migration	Maggioni et al. (2014)
Gastric cancer cells + nude mice (*in vitro* + *in vivo*)	Naringin (dose not specified)	Not reported	↓ proliferation; ↓ migration; ↓ invasion; ↓ EMT (Vimentin, Zeb1); ↑ E-cadherin; ↓ PI3K/Akt signaling; ↑ apoptosis; ↑ G0/G1 arrest	PI3K/AKT/Zeb1 pathway modulation; cell cycle arrest; EMT suppression	P-AKT, Zeb1, Vimentin, E-cadherin, flow cytometry, transwell assay	Zhu et al. (2023)
Esophageal CSC xenograft mice + cell model	300 μM (24 h)	354 μM	↓ systemic toxicity (DOX side effects); ↓ oxidative stress; ↓ tumor growth (combo therapy); ↑ body weight stability; ↑ antioxidant status (SOD, TAC); ↑ protective adjuvant effect	Cytoprotection & CSC preservation; ROS and oxidative stress modulation	MDA, TAC, SOD, SOX2, OCT4, Annexin/7-AAD, cell cycle (PI)	Tajaldini et al. (2020)
DMBA-induced hamster buccal pouch (*in vivo*)	**20 mg/kg b.w. (hesperetin suspension in 0.1% CMC; 1 mL/hamster)**	Not applicable	↓ glycoconjugates abnormalities; ↓ cytokeratin expression; ↓ hyperplasia/dysplasia progression; ↑ normalization of tissue biomarkers	Restoration of glycoconjugates & cytokeratin expression; epithelial integrity preservation	Protein-bound hexose, hexosamine, total sialic acid, fucose, PAS staining, immunohistochemistry	Babukumar et al. (2018)
MNNG gastric carcinogenesis (exosome-mediated)	5, 10, 25, 50, and 100 μM (CCK-8 screening for 96 h); 50 and 100 μM selected for subsequent experiments	Not reported	↓ exosome-driven proliferation; ↓ migration; ↓ invasion; ↓ EMT; ↓ stemness; ↑ miR-526b-5p targeting; ↓ circ0008274-mediated oncogenic signaling	Exosomal circ0008274 modulation; miR-526b-5p targeting; inhibition of EMT & stemness	circ0008274, miR-526b-5p, EMT markers, colony formation, transwell migration	Liang Z. F. et al. (2025)

miR, microRNA; lncRNA, long non-coding RNA; circRNA, circular RNA; ROS, reactive oxygen species; SOD, superoxide dismutase; CAT, catalase; GPx, glutathione peroxidase; TNF-α, tumor necrosis factor-alpha; IL-1β, interleukin-1, beta; IL-6, interleukin-6; NF-κB, nuclear factor kappa-light-chain-enhancer of activated B cells; Nrf2, nuclear factor erythroid 2–related factor 2; HO-1, heme oxygenase-1; Bax, BCL2-associated X protein; Bcl-2, B-cell lymphoma 2; Caspase-3, cysteine-aspartic protease-3; GCLC, glutamate-cysteine ligase catalytic subunit; GCLM, glutamate-cysteine ligase modifier subunit; LPS, lipopolysaccharide; HFD, high-fat diet; ALT, alanine aminotransferase; AST, aspartate aminotransferase; ALP, alkaline phosphatase; MTT, 3-(4,5-dimethylthiazol-2-yl)-2,5-diphenyltetrazolium bromide; HE, hematoxylin-eosin staining.

↑ increase/enhancement/restoration; ↓ decrease/inhibition; ↔ normalization/balance restored.

### 8.1 HET, NAR, and naringin: epigenetic and exosome-driven tumor regulation

One emerging area of interest is the ability of flavanones to influence epigenetic regulation. In GC models, HET promoted degradation of the histone methyltransferase DOT1L, resulting in reduced H3K79 methylation and decreased expression of metastasis-associated genes (Wang SW. et al., 2021). These molecular alterations were accompanied by reduced migration and invasion *in vitro* and diminished metastatic behavior in xenograft models, suggesting that epigenetic modulation may contribute to the antitumor activity of HET (Wang SW. et al., 2021). However, the relative contribution of DOT1L degradation to the overall antitumor activity of HET remains to be clarified. In OSCC cells, NAR induced cell-cycle arrest and reduced cellular proliferation without markedly inducing apoptosis, indicating that cytostatic mechanisms may substantially contribute to growth inhibition (Maggioni et al., 2014). Similar effects have been reported in GC models, where naringin suppressed proliferation, migration, and invasion while promoting cell-cycle arrest and modulating EMT-associated proteins (Zhu et al., 2023). Collectively, these findings suggest that flavanones suppress metastatic progression by coordinating cell-cycle regulation with partial reversal of EMT (Zhu et al., 2023; Maggioni et al., 2014). Although these findings are broadly consistent, differences in experimental conditions, concentrations, and model systems currently limit direct comparisons among studies.

Increasing evidence also suggests that flavanones may influence tumor progression through modulation of ncRNA signaling and exosome-mediated intercellular communication. In MNNG-induced gastric carcinogenesis models, HET reduced the oncogenic potential of tumor-derived exosomes and attenuated exosome-mediated proliferation, invasion, stemness, and EMT in recipient gastric epithelial cells (Liang ZF. et al., 2025). Mechanistically, these effects were associated with regulation of the circ008274/miR-526b-5p axis, highlighting a potential role for flavanones in modulating post-transcriptional regulatory networks involved in malignant progression (Liang ZF. et al., 2025). These findings identify ncRNA-dependent exosomal signaling as a promising mechanistic target for future investigation. Limited evidence from related upper gastrointestinal cancer models suggests that naringin may influence cancer stem cell (CSC)–associated pathways; however, the biological significance of these observations remains uncertain and requires validation in oral and gastric cancer models (Tajaldini et al., 2020). Beyond intracellular signaling, flavanones may also influence epithelial architecture and tissue integrity. In the DMBA-induced hamster buccal pouch model, HET restored alterations in cell surface glycoconjugates and normalized cytokeratin expression, accompanied by improvements in histopathological features, including reduced hyperplasia and dysplasia (Babukumar et al., 2018). These observations further support a role for flavanones in preserving epithelial differentiation during oral carcinogenesis (Babukumar et al., 2018). Overall, current evidence indicates that citrus flavanones regulate multiple aspects of tumor progression through coordinated modulation of epigenetic mechanisms, ncRNA signaling, EMT, and epithelial homeostasis. However, mechanistic evidence remains largely preclinical, and integrated multi-omics studies together with clinically relevant models are required to define the translational significance of these pathways in oral and gastric carcinogenesis.

### 8.2 Flavanone-mediated epigenetic and ncRNA regulation in tumor progression

Accumulating evidence suggests that the anticancer activity of flavanones extends beyond direct cytotoxicity and may involve modulation of epigenetic and ncRNA-dependent regulatory networks. Although most available studies have been conducted in cancer models other than oral cancer and GC, these investigations collectively indicate that flavanones can influence DNA methylation, histone modification, and miRNA-, long non-coding RNA (lncRNA)- and circular RNA (circRNA)-mediated signaling pathways, thereby affecting cellular proliferation, apoptosis, oxidative stress responses, and metastatic behavior (Table 4). Across diverse preclinical models, flavanones have been shown to regulate miRNAs involved in redox homeostasis, tumor suppression, and inflammatory signaling. For example, NAR and Hsp modulate tumor-associated miRNAs, including miR-17-3p, miR-34a, and miR-132, which are linked to antioxidant defense and regulation of proliferative signaling (Ibrahim et al., 2024; Tan et al., 2020; Curti et al., 2017). Flavanones may also influence lncRNA-mediated regulatory networks. Studies in non-oral and non-gastric cancer models indicate that NAR and HET can modulate lncRNA–miRNA interactions, including XIST- and H19-associated pathways, thereby affecting apoptosis, migration, and invasion-related phenotypes (Abdallah et al., 2022; Wen et al., 2023). However, the relevance of these mechanisms to OSCC and GC remains largely unexplored. Beyond ncRNA regulation, flavanones may influence broader epigenetic processes, including DNA methylation and chromatin-associated regulation. Experimental studies have reported alterations in methylation patterns, tumor-associated miRNA profiles, and circRNA-related inflammatory signaling following flavanone exposure (Fernández-Bedmar et al., 2017; A’laa et al., 2024; Magura et al., 2022; Ji et al., 2022; Sun et al., 2024). Nevertheless, the causal contribution of these epigenetic changes to oral and gastric cancer prevention remains uncertain. These studies collectively support the concept that flavanones can influence multiple layers of epigenetic regulation; however, the extent to which these mechanisms contribute directly to oral and GC chemoprevention remains unclear. Despite encouraging findings, several important limitations should be acknowledged. Most mechanistic evidence originates from non-oral and non-gastric models, limiting direct translational relevance. In addition, heterogeneity in flavanone exposure, experimental systems, and ncRNA targets complicates cross-study comparisons. Overall, available preclinical evidence suggests that flavanones, particularly NAR, HET, and Hsp, may modulate tumor progression through complementary epigenetic and ncRNA-dependent regulatory pathways involving DNA methylation, histone-associated signaling, and miRNA/lncRNA/circRNA networks. These regulatory effects may influence tumor-associated processes, including proliferation, cellular plasticity, and metastatic potential. Nevertheless, future studies specifically focusing on oral and gastric carcinogenesis are required to determine which of these epigenetic alterations are causally involved in chemoprevention and whether they can be translated into clinically relevant biomarkers or therapeutic targets.

**TABLE 4:** Supportive studies on flavanone-mediated epigenetic and non-coding RNA regulation across diverse preclinical models.

Model	Dose/Concentration	IC_50_	Findings	Mechanistic/Molecular insights	Functional assays/Measurements	References
Caco-2 cells (*in vitro*)	1–1,000 μg/mL (24 h); 1, 10, and 100 μg/mL for mechanistic assays	Not reported	↓ miR-17-3p, ↓ miR-25-5p; ↑ MnSOD, ↑ GPx2; ↑ endogenous antioxidant defense; ↔ inflammatory mRNA mismatch (TNF-α, IL-6 not fully correlated)	Downregulation of miR-17-3p and miR-25-5p; upregulation of target mRNAs coding for antioxidant enzymes (MnSOD, GPx2); partial regulation of inflammatory cytokines	Real-time PCR for miRNAs and target mRNAs	Curti et al. (2017)
HL60 leukemia cells (*in vitro*)	5, 10, 20, 40, and 80 μM tested in HL60/KG-1 AML cells for 24 h; 10 μM (low dose) and 40 μM (high dose) selected for mechanistic experiments	Not reported	↑ apoptosis; ↓ lncRNA XIST; ↑ miR-34a; ↓ HDAC1; activation of XIST/miR-34a/HDAC1 axis leading to cell death	Decreased lncRNA XIST and HDAC1 expression; increased miR-34a; XIST/miR-34a/HDAC1 signaling axis	Flow cytometry, apoptosis assays, sh-XIST transfection, miR-34a modulation	Wen et al. (2023)
HL60 cells + DEN rat HCC model (*in vitro* + *in vivo*)	Hesperidin 250–1,000 ppm	IC_50_ = 12.5 mM	↓ DNA methylation (LINE-1, ALU); ↓ tumor nodules; ↓ proliferation; ↑ epigenetic hypomethylation; ↑ chemopreventive effect	Dose-dependent cytotoxicity; DNA hypomethylation of LINE-1 (47%) and ALU-M2 (32%); modulation of oncogenic and tumor-suppressive microRNAs	MTT assay, methylation analysis, *in vivo* hepatic nodule count, histopathology	Fernández-Bedmar et al. (2017)
DEN/CCl4-induced HCC rats (*in vivo*)	Hesperidin 150 mg/kg	Not determined	↓ miR-221; ↓ FGF-2; ↓ oxidative stress; ↑ Nrf2; ↑ caspase-3; ↑ apoptosis; ↓ DNA hypermethylation; ↑ antiangiogenic effects	Global DNA hypo-methylation; downregulation of miR-221, FGF-2; upregulation of Nrf2, Bcl-2, caspase-3	Serum ALT, AST, ALP; liver histopathology; qPCR for miRNAs	A’laa et al. (2024)
Breast cancer cells (MDA-MB-231, MCF-7)	Hesperetin (dose not specified)	Not reported	↓ H19 lncRNA; ↑ miR-486-5p; ↓ ICAM-1; ↓ migration, invasion, proliferation; ↑ anti-metastatic signaling	Modulation of lncRNA H19 / miR-486-5p axis; ICAM-1 downregulation; ncRNA-mediated metastatic control	qRT-PCR, MTT, colony formation, migration & invasion assays	Abdallah et al. (2022)
A549 and H460 lung cancer cells (*in vitro*)	100–1,000 µM (48 h)	814.36 µM (A549); 944.21 µM (H460)	↓ proliferation; ↓ migration; ↑ miR-34a; ↓ NF-κB signaling; ↑ apoptosis; ↑ p53; ↓ PD-L1, ↓ EGFR; cell cycle arrest (sub-G1, G2/M)	Upregulation of miR-34a (∼2.3-fold) & miR-132 (∼2.1-fold); downregulation of PD-L1 & ZEB2; NF-κB pathway suppression	Flow cytometry, MTT, wound healing, colony formation, xenograft tumor volume	Ibrahim et al. (2024)
NSCLC cells + rat model (*in vitro* + *in vivo*)	1 and 2.5 µM (48 h, *in vitro*); 60 mg/kg/day (*in vivo*)	Not reported	↑ miR-132; ↓ ZEB2; ↓ proliferation; ↑ apoptosis; ↓ colony formation; ↓ tumor volume; ↑ anti-metastatic signaling	miR-132 upregulation; ZEB2 inhibition	MTT, flow cytometry, luciferase assays, xenograft tumor volume	Tan et al. (2020)
C57BL/6 mice (LPS-induced liver injury), C57BL/6 mice (high-fat diet-induced obesity/liver inflammation), AML-12 mouse hepatocytes	30 mg/kg/day, oral gavage for 7 days, 100 mg/kg/day, oral gavage for 8 weeks, 50 μg/mL (≈183.6 μM), 12 h treatment	Not reported	↓ oxidative stress; ↓ inflammation (TNF-α, IL-1β, IL-6, MCP1); ↑ antioxidant genes (GCLC, GCLM); regulation of lncRNA–mRNA axis; ↓ liver dysfunction; ↑ tissue protection	Upregulation of antioxidant enzymes (GCLC 2.2-fold, GCLM 1.9-fold); downregulation of inflammatory factors (MCP1, TNF-α, IL-1β, IL-6); 36 lncRNAs and 603 mRNAs modulated	Transcriptome sequencing, qPCR, cytokine measurements	Ji et al. (2022)
LPS-induced acute liver injury in mice + RAW264.7 cells	25, 50, and 100 mg/kg/day (oral gavage, administered 24 h before LPS challenge)	Not reported	↓ circDcbld2; ↓ JAK2/STAT3 signaling; ↓ inflammation; ↓ liver injury markers; ↓ inflammatory cytokines; ↑ hepatoprotection	Downregulation of circDcbld2; suppression of JAK2/STAT3 pathway	qPCR, liver function tests (ALT, AST, ALP), histopathology	Sun et al. (2024)
MCF-7 breast cancer cells (*in vitro*)	Hesperidin + luteolin (isolated compounds)	Not reported	↓ cell viability; ↑ apoptosis; ↑ caspase-3/8/9; ↓ Bcl-2; ↑ Bax; ↑ G0/G1 & sub-G1 arrest; ↓ miR-21; ↑ miR-16; ↑ miR-34a	Apoptotic microRNA modulation (up miR-16/-34a, down miR-21); regulation of cell cycle and apoptosis genes (Bcl-2, Bax, caspases)	MTT, flow cytometry, qPCR for miRNAs & apoptotic genes	Magura et al. (2022)

miR, microRNA; lncRNA, long non-coding RNA; circRNA, circular RNA; ROS, reactive oxygen species; MnSOD, manganese-dependent superoxide dismutase; GPx2, glutathione peroxidase 2; TNF-α, tumor necrosis factor-alpha; IL-6, interleukin-6; HDAC1, histone deacetylase 1; XIST, X-inactive specific transcript; IC50, half maximal inhibitory concentration; DEN, diethyl nitrosamine; CCl4, carbon tetrachloride; ALT, alanine aminotransferase; AST, aspartate aminotransferase; ALP, alkaline phosphatase; Nrf2, nuclear factor erythroid 2–related factor 2; FGF-2, fibroblast growth factor-2; Bcl-2, B-cell lymphoma 2; Caspase-3, cysteine-aspartic protease-3; ICAM-1, intercellular adhesion molecule-1; PD-L1, programmed death-ligand 1; ZEB2, zinc finger E-box binding homeobox 2; NF-κB, nuclear factor kappa-light-chain-enhancer of activated B cells; GCLC, glutamate-cysteine ligase catalytic subunit; GCLM, glutamate-cysteine ligase modifier subunit; MCP1, monocyte chemoattractant protein-1; LPS, lipopolysaccharide; AML12, alpha mouse liver 12 cells; HD-2a, hesperetin derivative 2a; MTT, 3-(4,5-dimethylthiazol-2-yl)-2,5-diphenyltetrazolium bromide; HE, hematoxylin-eosin staining.

↑ increase/enhancement/restoration; ↓ decrease/inhibition; ↔ normalization/balance restored.

## 9 Additional mechanistic pathways: aryl hydrocarbon receptor (AhR), peroxisome proliferator-activated receptor gamma (PPARγ), and nuclear receptor 4A1 (NR4A1) signaling

Beyond canonical antioxidant and kinase-dependent pathways, citrus flavanones may also influence nuclear receptor signaling networks involved in inflammation, metabolism, and carcinogenesis. Notably, the AhR, PPARγ, and orphan NR4A1 have been identified as plausible mechanistic hubs that forge connections between dietary flavonoids and the modulation of inflammation, metabolism, and carcinogenesis. AhR is a ligand-activated transcription factor involved in xenobiotic metabolism, immune regulation, and epithelial homeostasis. Flavonoids can modulate AhR activity, influencing downstream inflammatory and oxidative stress-related pathways; however, its biological consequences are highly context dependent, with potential tumor-promoting or tumor-suppressive effects depending on cellular conditions (Denison and Nagy, 2003; Safe et al., 2018; Goleij et al., 2025; Park et al., 2022; Goya-Jorge et al., 2021; Yang et al., 2019; Nishiumi et al., 2011). PPARγ represents another potential mediator of flavanone activity by regulating lipid metabolism, inflammation, and cellular differentiation. Flavonoid-mediated PPARγ activation has been associated with reduced inflammatory signaling, altered metabolic programming, and inhibition of tumor-associated phenotypes, although direct evidence in oral and gastric carcinogenesis remains limited (Lehrke and Lazar, 2005; Chi et al., 2021). NR4A1 (Nur77) has recently emerged as another flavonoid-responsive nuclear receptor involved in apoptosis, mitochondrial regulation, and oncogenic signaling. Similar to AhR, NR4A1 exhibits context-dependent functions, and its contribution to flavanone-mediated cancer prevention or therapy requires further clarification (Goleij et al., 2025; Safe and Karki, 2021; Shrestha et al., 2021; Lee et al., 2023; Wang H. et al., 2026; Xu et al., 2025; Zhang et al., 2023). This duality is consistent with the pleiotropic characteristics of flavanone activity demonstrated in various preclinical models. Collectively, AhR, PPARγ, and NR4A1 signaling may represent additional regulatory layers through which citrus flavanones influence cancer-related processes. Integration of these nuclear receptor pathways with established signaling networks highlights the pleiotropic nature of flavanone actions. However, their specific relevance to human oral and gastric carcinogenesis remains largely unexplored and requires validation in clinically relevant models.

## 10 Comparative mechanisms of NAR/Naringin and HET/Hsp in OSCC and GC

Overall, available evidence indicates that NAR/naringin and HET/Hsp influence overlapping but partially distinct carcinogenesis-related processes in OSCC and GC models. Nevertheless, current findings suggest potential differences in their predominant molecular actions. NAR and naringin have been frequently associated with modulation of intracellular stress responses, cell-cycle control, and PI3K/AKT-related survival signaling, whereas HET and Hsp have additionally been investigated for their effects on epigenetic regulation, ncRNA networks, immune signaling, and TME-associated processes. However, direct comparative studies remain limited, and heterogeneity in models, dosing strategies, and outcome measures prevents conclusions regarding the relative superiority of individual flavanones.

## 11 Limitations

Although preclinical studies support the biological relevance of dietary flavanones, including NAR, naringin, HET, and Hsp, several limitations restrict their translation into clinical applications. A major limitation is the predominant reliance on *in vitro* systems and animal models, including chemically induced OSCC and GC models. While these models reproduce selected cancer-related features, they do not fully capture human tumor complexity, including immune heterogeneity, systemic metabolism, stromal interactions, and patient variability. In addition, substantial heterogeneity exists across studies regarding flavanone concentrations, treatment duration, formulations, and experimental endpoints, limiting direct comparisons and mechanistic interpretation. Pharmacokinetic limitations represent another major barrier. Poor solubility, limited bioavailability, and extensive metabolism reduce systemic exposure, whereas the clinical relevance, optimal dosing, and long-term safety of nanoparticle-based formulations remain uncertain. Furthermore, flavanones exert pleiotropic biological effects through interconnected molecular networks; however, the relative importance of individual pathways and their integration within human tumors remain poorly defined, particularly in the context of the dynamic TME. Similarly, evidence regarding immune modulation, angiogenesis, and microbiota-associated effects remains largely preclinical, and their relevance in human OSCC and GC requires validation. Finally, long-term safety data and well-designed clinical trials remain limited. Differences in diet, genetics, metabolism, and comorbid conditions may further influence efficacy and safety, highlighting the need for standardized translational studies. Overall, while preclinical findings support the biological potential of flavanones, substantial gaps remain in pharmacokinetics, mechanistic integration, and clinical validation. Carefully designed clinical investigations are therefore required before these compounds can be incorporated into cancer prevention or adjunctive therapeutic strategies.

## 12 Future directions

Despite substantial preclinical evidence supporting the potential of citrus flavanones in OSCC and GC models, several gaps currently limit clinical translation. Future studies should prioritize physiologically relevant models that better capture chronic exposure, tumor heterogeneity, and host-specific responses. Advanced models, including patient-derived organoids and genetically relevant systems, may provide more predictive platforms for evaluating inter-individual responses and identifying biomarkers of flavanone sensitivity. A major translational challenge remains optimization of delivery systems and pharmacokinetics. Although nanoformulations improve stability and bioavailability in experimental models, clinically relevant studies are needed to define dosing, tissue targeting, release profiles, and long-term safety. Future investigations should also clarify how flavanones influence tumor–host interactions, including immune regulation, angiogenesis, and microbiota-associated pathways, using immunocompetent models that better reproduce human OSCC and GC biology. In addition, combinations with standard anticancer treatments require systematic evaluation to identify synergistic effects and clinically relevant dosing strategies. Integration of multi-omics approaches may further improve mechanistic understanding and biomarker discovery. Finally, consideration of dietary patterns, microbiome composition, and genetic variability will be important for defining personalized approaches and explaining differences in flavanone responses. Collectively, these efforts are required to bridge the gap between promising preclinical findings and clinical application of citrus flavanones in cancer prevention and therapy.

## 13 Conclusion

Dietary flavanones, notably NAR, naringin, HET, and Hsp, represent pleiotropic bioactives that influence OSCC and GC progression through coordinated regulation of multiple biological layers rather than isolated molecular targets. Across preclinical models, these compounds influence multiple cancer-associated processes, while their effects extend beyond tumor cells to regulatory networks involved in epigenetic control and the TME. Importantly, this review integrates intracellular signaling, epigenetic regulation, ncRNA networks, TME remodeling, and delivery strategies into a unified framework, providing a systems-level perspective beyond conventional flavonoid-focused analyses. Current evidence suggests that NAR/naringin are frequently associated with cell-intrinsic regulatory mechanisms, whereas HET/Hsp have additionally been investigated for broader effects involving epigenetic regulation, ncRNA networks, immune signaling, and intercellular communication. This distinction supports the concept that flavanones act as context-dependent modulators of tumor biology rather than single-pathway inhibitors. Beyond tumor-cell intrinsic effects, flavanones may influence the TME by modulating immune surveillance, inflammatory signaling, angiogenic processes, and tumor–host interactions. Nanoformulations represent a promising strategy to overcome bioavailability limitations and enhance translational feasibility. Although the current evidence base remains predominantly preclinical, convergence across independent studies supports a multi-level model in which flavanones influence tumor biology through coordinated intracellular and microenvironmental regulation. However, heterogeneity in experimental models, dosing approaches, and delivery systems continues to limit clinical extrapolation. Future research should prioritize clinically relevant validation models, optimized delivery strategies, and identification of effective therapeutic windows to facilitate translation of citrus flavanones into adjunctive approaches for OSCC and GC prevention and management.
